# Visual hallucinations in Alzheimer's disease is significantly associated with clinical diagnostic features of dementia with Lewy bodies

**DOI:** 10.1371/journal.pone.0186886

**Published:** 2017-10-31

**Authors:** Pai-Yi Chiu, Min-Hsien Hsu, Chein-Wei Wang, Chun-Tang Tsai, Ming-Chyi Pai

**Affiliations:** 1 Department of Neurology, Show Chwan Memorial Hospital, Changhua, Taiwan; 2 Department of Neurology, Lin-Shin Hospital, Taichung, Taiwan; 3 Department of Guidance and Counseling, National Changhua University of Education, Changhua, Taiwan; 4 Division of Behavioral Neurology, Department of Neurology, National Cheng Kung University Hospital, College of Medicine, National Cheng Kung University, Tainan, Taiwan; 5 Alzheimer’s Disease Research Center, National Cheng Kung University Hospital, Tainan, Taiwan; 6 Institute of Gerontology, College of Medicine, National Cheng Kung University, Tainan, Taiwan; Oslo Universitetssykehus, NORWAY

## Abstract

Visual hallucinations (VHs) are among the most striking features of dementia with Lewy bodies (DLB). Given that Lewy body pathology is frequently observed in the brains of patients with AD, we aimed to study factors associated with VHs in AD and examine their association with DLB features. This cross-sectional study enrolled a consecutive series of AD patients who visited the dementia clinic of a regional hospital. Clinically diagnosed possible or probable DLB cases were excluded. VH frequency and associated factors including age, sex, education, disease severity, DLB features, vascular risk factors, cognitive function, and neuropsychiatric symptoms were compared between AD patients with VHs (VH+) and those without VHs (VH−). Among a total of 295 patients analyzed, 42 (14.2%) had VHs. After adjusting for age, sex, and disease severity, DLB features including fluctuations in cognition scores, rapid-eye-movement behavioral disorder (RBD), and severe neuroleptic sensitivity were more frequent in the VH+ group. Furthermore, depression score, total Neuropsychiatric Inventory (NPI) score, and total caregiver burden score as assessed by the NPI were higher in the VH+ group. Among neuropsychiatric symptoms, delusions, hallucinations in the non-visual domains, anxiety, and disinhibition were more frequent in the VH+ group. Conversely, none of the vascular risk factors (VRFs) or cognitive domains of the Cognitive Abilities Screening Instrument (CASI) was associated with VHs in AD. In summary, VHs, albeit occurring at a low rate, had a high impact on AD. Diagnostic features of DLB, including fluctuations, RBD, and severe neuroleptic sensitivity were significantly associated with VHs in AD. AD patients with VHs tended to have more severe neuropsychiatric symptoms and greater caregiver burden.

## Introduction

Alzheimer’s disease (AD) is the most common type of dementia. Psychotic symptoms, including delusions and hallucinations, are striking features that cast a great impact on both patients with AD (PwAD) and their caregivers [1]. Among psychotic symptoms, delusions start in early stages of AD and are frequently comorbid throughout the course of dementia [2,3], whereas visual hallucinations (VHs), which are far less frequently observed in PwAD, manifest in later disease stages [2]. Distinct from AD, VHs are among the most important diagnostic features for dementia with Lewy bodies (DLB) [4,5]. VHs may start earlier in DLB and are observed significantly more frequently in DLB than in AD or other dementias [2,6–8].

Previous studies reveal that dementia patients with psychotic features are usually older and that disease onset occurs at a later age [9,10]. Additionally, dementia is usually more severe in patients with psychotic features [9–11]. Gender association with VH symptoms, in contrast, has been noted or studied less in previous studies [11,12]. Impaired visual acuity, perception, and/or visuospatial dysfunction are also associated with VHs in AD [12,13]. For example, the presence of VHs in PwAD was associated with increased occipital periventricular hyperintensities and an absence of occipital deep white matter hyperintensities on brain magnetic resonance imaging (MRI) [14]. Hallucinations in PwAD are significantly associated with depression, a relationship that is further mediated by inhibition [15]. Moreover, hallucinations are significantly more likely to occur in subjects with no *APOE4* alleles than in those with two E4 alleles [16]. AD patients with VHs are also more likely to have Lewy body pathology than those without VHs [17]. Psychosis of AD leads to a faster functional impairment and increases the mortality risk [18]. The presence of hallucinations is selectively associated with more rapid cognitive decline in AD [19]. Furthermore, hallucinations are associated with a 78% increase in risk of death in AD [20].

Whereas there is robust evidence on the identity of factors associated with VHs, few studies examined clinical features of DLB such as cognitive fluctuation, parkinsonism, rapid-eye movement sleep behavior disorder (RBD), and severe neuroleptic sensitivity in AD patients with VHs. Lewy body pathology is frequently observed in AD [21] and in AD with VHs [17], and there is clinical evidence of higher rates of RBD in AD patients with hallucinations as compared to those without hallucinations [22]. Therefore, in this study, we investigated whether VHs in AD were associated with higher frequencies of clinical features of DLB. Furthermore, we assessed possible associations of additional clinical factors including age, education, disease severity, cognitive function, neuropsychiatric symptoms, vascular risk factors (VRFs), and medication among AD patients with VHs and those without VHs.

## Methods

### Participants

A consecutive series of participants visiting the dementia clinic of a regional hospital between September 2009 and June 2013 were enrolled in this cross-sectional study [23]. Diagnosis of dementia was reached according to the criteria for primary degenerative dementia defined in the fourth edition of the Diagnostic and Statistic Manual of Mental Disorders (DSM-IV). AD diagnosis was based on to the criteria for probable AD defined in the National Institute of Neurological and Communicative Disorders and Stroke and the Alzheimer’s Disease and Related Disorders Association (NINCDS-ADRDA) [24], which are widely used, highly reliable criteria that are validated for clinical diagnosis of AD [25]. All patients received at least one cerebral computed tomography or cerebral MRI and blood screening tests to rule out other potential etiologies of cognitive decline. Patients with uncorrected dysfunctions of vision or hearing that could significantly inhibit compliance with the instructions and procedures used in clinical tests were excluded in this study. The following data were used in this study: 1) age, sex, education, disease duration, VRFs, and current medication; 2) dementia severity based on the Clinical Dementia Rating Scale (CDR) [26], CDR scale sum of boxes (CDR-SB) score, and depression severity based on the Hamilton Depression Rating Scale (HDRS) [27]; 3) clinical DLB features including cognitive fluctuation, parkinsonism, VHs, RBD, and severe neuroleptic sensitivity; 4) Mini-Mental State Examination (MMSE) [28] and cognitive performance on the Cognitive Abilities Screening Instrument, Chinese version 2.0 (CASI C-2.0) [29]; 5) neuropsychiatric symptoms included in the twelve-item version of the Neuropsychiatric Inventory (NPI) based on observations within the month prior to study enrollment [30,31].

### Assessment of clinical features and exclusion of DLB

At the dementia clinic, all patients and their primary caregivers were interviewed by a neurologist and a trained psychologist for dementia diagnosis and assessment of diagnostic features. Diagnosis of cognitive fluctuation was based on a clinical history of fluctuation in cognition or a Mayo Fluctuation Composite Score (MFCS) of >2. Diagnosis of parkinsonism was based on the presence of at least two of the following criteria: bradykinesia, tremor, rigidity, and postural instability. Diagnosis of RBD was based on the presence of the minimal criteria for RBDs as outlined in the International Classification of Sleep Disorders (ICSD) [32]. Severe neuroleptic sensitivity was diagnosed based on the clinical history for both the use of neuroleptic drugs and the presence of an obvious association of adverse events with the neuroleptic drug. In patients presenting with any one of the core features (cognitive fluctuation, VHs, parkinsonism) of the DLB diagnosis, three steps were taken to rule out the possibility of probable or possible DLB. First, the patient had to fulfill the criteria for a typical AD presentation as established by the NIA-AA [33]. Second, in those patients with parkinsonism, the onset of dementia had to precede the onset of parkinsonism by at least two years to be eligible for inclusion in final analysis. Third, among patients with no symptoms of parkinsonism, those who still were deemed to fulfill the criteria for probable or possible DLB were excluded from final analysis.

### Assessment of VHs and other neuropsychiatric symptoms

All patients and their primary caregivers were interviewed by a trained neuropsychologist for the assessment of VHs and other neuropsychiatric symptoms included in the NPI. The NPI is a validated, standardized, and widely used instrument that is developed specifically for neuropsychiatric symptoms of dementia [30]. All twelve NPI subscales were rated using symptom frequency ranging from 1 (occasional) to 4 (very frequent), symptom severity ranging from 1 (mild) to 3 (severe), and caregiver burden from 0 (none) to 5 (extreme) [31].

### Assessment of disease severity and cognitive function

Global dementia severity was assessed according to the CDR and CDR-SB scores. Cognitive functions were assessed with the MMSE and CASI C-2.0. Cognitive tests for all patients were performed by a trained neuropsychologist. Dementia and its subtype were determined by a consensus meeting composed of two neurologists, one geriatric psychiatrist, and a neuropsychologist.

### Data analysis

The Chinese version of SPSS 19.0 for Windows (IBM, SPSS Inc., Chicago) was used for statistical analyses. Comparisons between VH+ and VH− PwAD groups based on age, education, disease durations, MMSE, MFCS, HDRS, total and subscale CASI C-2.0 scores, and composite scores (frequency × severity) of overall NPI as well as NPI subscales were analyzed using independent *t* test. Sex, CDR score, VH frequency, clinical features, frequency of each of the twelve NPI subscales, and VRFs were analyzed using the chi-square or Fisher’s exact test. To examine associations between demographics, cognitive performance, neuropsychiatric symptoms, and motor function among psychosis and non-psychosis PwAD groups, multiple logistic regression model was used, and odd ratios (ORs) were derived from a stepwise approach that considered age, disease severity, sex, and interested factors.

### Ethical consideration

This study was reviewed by the Committee for Medical Research Ethics of Lin-Shin Hospital and approved by the Data Inspectorate. All participants provided informed consent for participation in this study. In patients with mild disease, informed consent was provided by the patient themselves. In patients with moderate to severe disease, informed consent was provided by the primary caregiver of the patient.

## Results

A total of 295 patients who fulfilled the criteria for AD and completed the study were analyzed. In this cohort, there were 204 (69.2%) female patients. Table 1 shows the comparison of demographic and background characteristics among PwAD with VHs (n = 42, 14.2%) and those without VHs (n = 253, 85.8%). Within the VH+ group, the frequency of VHs of non-family people (59.7%) was highest, followed by VHs of relatives (42.9%), living families (35.7%), children (9.4%), animals/insects (9.4%), and others (9.4%). Before adjustment, VHs in PwAD were associated with older age, more advanced dementia as assessed by CDR and CDR-SB scores, worse cognitive performance as assessed by MMSE and CASI scores, and more severe neuropsychiatric disturbances as assessed by total NPI score. After adjustment for age, sex, and disease severity, comparison of the VH+ and VH− groups revealed that the VH+ group had a higher total NPI score (OR, 1.09; *p* < 0.001), a higher total caregiver burden score (OR, 1.17; *p* < 0.001) by the NPI, a higher MFCS score (OR, 1.65; *p* < 0.001), and a higher HDRS score (OR, 1.07; *p* = 0.027).

**Table 1:** Demographic and background characteristics of patients with Alzheimer’s disease (AD) with or without visual hallucinations (VHs).

	Mean (SD, range)		Non-adjusted		Adjusted	
	VH+	VH−	t/χ^2^	*p*	OR (95% CI)	*p*
N	42	253				
Age, years	81.4 (7.9, 54–95)	77.3 (7.8, 60–100)	**3.16**	**0.002**	NA	
Sex, f/m	27/15	177/76	0.544	NS	NA	
Education, years	5.2 (4.7, 0–16)	5.2 (4.8, 0–16)	−0.14	NS	0.99 (0.91–1.07)	NS
Dementia duration, years	4.2 (4.3, 2–21)	3.3 (2.6, 1–21)	1.86	NS	0.97 (0.87–1. 08)	NS
CDR 0.5/1/2-3	2/23/17	92/132/29	**30.78**	**<0.001**	NA	
CDR-SB	8.5 (2.9, 3.0–13.0)	5.5 (2.7, 2.0–14.0)	**6.37**	**<0.001**	0.61 (0.82–1.41)	NS
MMSE	14.8 (7.6, 0–26)	18.6 (6.0, 0–28)	**−3.64**	**<0.001**	1.00 (0.98–1.02)	NS
CASI	46.7 (25.6, 0–82)	60.7 (19.9, 2–91)	**−4.06**	**<0.001**	0.98 (0.96–1.00)	NS
NPI	26.9 (14.3, 6–57)	13.9 (10.4, 0–44)	**7.05**	**<0.001**	**1.09 (1.06–1.12)**	**<0.001**
NPI burden	15.2 (7.8, 0–32)	7.8 (6.1, 0–26)	**7.03**	**<0.001**	**1.17 (1.10–1.23)**	**<0.001**
HDRS	11.1 (6.3, 1–25)	5.5 (4.2, 0–20)	**2.17**	**0.031**	**1.07 (1.01–1.13)**	**0.027**
MFCS	1.8 (1.2, 0–4)	1.0 (1.2, 0–4)	**4.42**	**<0.001**	**1.65 (1.26–2.17)**	**<0.001**
Dementia drugs, n (%)	14 (33.3)	89 (35.2)	0.05	NS	0.91 (0.43–1.92)	NS
Antipsychotics, n (%)	7 (16.7)	15 (5.9)	**6.02**	**0.014**	2.12 (0.77–5.87)	NS

N, number of cases; NA, not applicable; NS, not significant; OR, odds ratio; CDR, Clinical Dementia Rating scale; CDR-SB, sum of boxes of the CDR; MMSE, Mini-Mental State Examination; CASI: Cognitive Abilities Screening Instrument; NPI: total score of the twelve-domain Neuropsychiatric Inventory; NPI burden, total caregiver burden scale in the NPI; HDRS, Hamilton Depression Rating Scale; MFCS, Mayo Fluctuation Composite Score; CI, confidence interval; SD, standard deviation. Variables were adjusted for age, sex, and disease severity.

Table 2 shows the clinical manifestations of PwAD with or without VHs. After adjustment for age, sex, and disease severity, comparison between the VH+ and VH− groups revealed that the VH+ group had higher rates of RBD (OR, 6.89; *p* < 0.001) and severe neuroleptic sensitivity (OR, 3.66; *p* = 0.040). None of the VRFs was significantly associated with VHs in PwAD.

**Table 2:** Clinical manifestation of patients with Alzheimer’s disease presenting with or without visual hallucinations (VHs).

	N (%)		Non-adjusted		Adjusted	
	VH+	VH−	t/χ^2^	*p*	OR (95% CI)	*p*
N	42	253				
DLB features						
Fluctuation, n (%)	13 (31.0)	28 (11.1)	**11.90**	**0.001**	2.03 (0.89–4.66)	NS
VH	42	0				
Parkinsonism, n (%)	10 (23.8)	42 (16.6)	1.29	NS	1.11 (0.48–2.55)	NS
RBD, n (%)	11 (26.2)	15 (5.9)	**18.40**	**<0.001**	**6.89 (2.50–19.0)**	**<0.001**
Severe neuroleptic						
sensitivity, n (%)	6 (14.3)	7 (2.8)	**11.35**	**0.001**	**3.66 (1.06–12.57)**	**0.040**
History of VRF						
Hypertension	19 (45.2)	128 (50.6)	0.41	NS	0.73 (0.37–1.43)	NS
Diabetes	10 (23.8)	52 (20.6)	0.23	NS	0.60 (0.27–1.37)	NS
Coronary artery disease	0 (0.0)	12 (4.7)	*	NS		
Hyperlipidemia	3 (7.1)	13 (5.1)	0.28	NS	0.24 (0.04–1.45)	NS
Arrhythmia	1 (2.4)	11 (4.3)	*	NS	2.16 (0.26–17.77)	NS
Cerebrovascular disease	7 (16.7)	27 (10.7)	1.27	NS	0.71 (0.28–1.83)	NS

DLB, dementia with Lewy bodies; AD, Alzheimer’s disease; N, number of cases; NA, not applicable; NS, not significant; OR, odds ratio; CI, confidence interval. RBD, rapid-eye-movement sleep behavior disorder. Adjusted risk estimates (ORs) of variables were adjusted for age, sex, and disease severity

* Fisher’s exact test.

**Fig 1:**
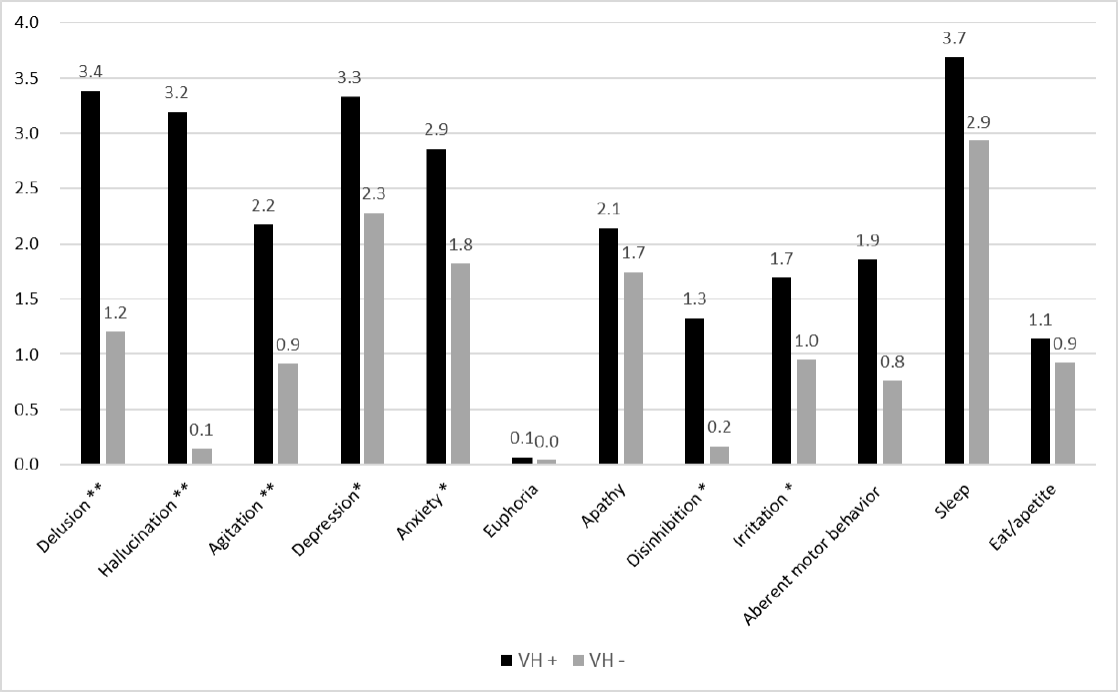
Comparison of the subscale scores in the twelve-item NPI between the VH+ and VH− AD groups. AD, Alzheimer’s disease; VH, visual hallucination. * *p* < 0.05; ** *p* < 0.005.

Fig 1 shows the comparison of the twelve-item NPI subscale scores between the VH+ and VH− groups. Briefly, PwAD in the VH+ group had significantly higher subscale scrores in delusions (t, 5.54; df, 293; *p* < 0.001), hallucinations (t, 19.28; df, 293; *p* < 0.001), agitation (t, 3.75; df, 293; *p* < 0.001), depression (t, 2.53; df, 293; *p* = 0.012), anxiety (t, 2.67; df, 293; p = 0.008), disinhibition (t, 5.18; df, 293; *p* < 0.001), irritation (t, 2.34; df, 293; *p* = 0.020), and aberrant motor behavior (t, 3.20; df, 293; *p* = 0.002).

After adjusting for age, sex, and disease severity, comparison of the cognitive function between the VH+ and VH− groups revealed that none of the cognitive domains in CASI was associated with VHs in AD. Table 3 shows the frequencies of all neuropsychiatric symptom in the NPI in the VH+ and VH− groups. After adjusting for age, sex, and disease severity, PwAD in the VH+ group had higher frequencies of delusion (OR, 6.37; *p* < 0.001), hallucinations in the non-visual domains (OR, 29.54; *p* < 0.001), anxiety (OR, 2.70; *p* = 0.011), and disinhibition (OR, 6.96; *p* < 0.001).

**Table 3:** Two models of risk estimates (odd ratios) for frequencies of neuropsychiatric symptoms in the NPI between the VH+ and VH− AD groups.

	Mean (SD, range)		Model 1		Model 2	
	VH +	VH−	OR (95% CI)	*p*	OR (95% CI)	*p*
N	42	253				
Delusion	32 (76.2)	73 (28.9)	**6.17 (2.74–13.89)**	**<0.011**	**6.37 (2.80–14.50)**	**<0.001**
Hallucination (non-VH)	23 (54.8)	9 (3.6)	**29.27 (10.81–79.28)**	**<0.001**	**29.54 (10.66–81.89)**	**<0.001**
Agitation	20 (47.6)	54 (21.3)	**2.19 (1.06–4.51)**	**0.034**	1.99 (0.93–4.25)	NS
Depression	30 (71.4)	144 (56.9)	1.92 (0.89–4.13)	NS	1.94 (0.89–4.24)	NS
Anxiety	28 (66.7)	116 (45.8)	**2.69 (1.26–5.74)**	**0.011**	**2.70 (1.26–5.82)**	**0.011**
Euphoria	1 (2.4)	2 (0.8)	2.15 (0.17–27.83)	NS	2.26 (0.17–30.50)	NS
Apathy	20 (47.6)	101 (39.9)	0.76 (0.37–1.55)	NS	0.76 (0.37–1.56)	NS
Disinhibition	12 (28.6)	10 (4.0)	**7.45 (2.78–20.01)**	**<0.001**	**6.96 (2.57–18.81)**	**<0.001**
Irritation	15 (35.7)	66 (26.1)	1.26 (0.60–2.63)	NS	1.22 (0.58–2.59)	NS
Aberrant motor behavior	13 (31.0)	41 (16.2)	1.40 (0.62–3.15)	NS	1.37 (0.60–3.14)	NS
Sleep	34 (81.0)	163 (64.4)	1.64 (0.68–3.94)	NS	1.61 (0.67–3.88)	NS
Eat/Appetite	11 (26.2)	55 (21.7)	1.04 (0.45–2.38)	NS	1.12 (0.48–2.62)	NS

AD, Alzheimer’s disease; NPI, Neuropsychiatric Inventory; NS, not significant; VH, visual hallucination; SD, standard deviation. Odds ratios (ORs) and 95% confidence intervals (CIs) were calculated using the non-psychosis group as reference. Model 1: ORs were adjusted for age and disease severity; Model 2: ORs were adjusted for age, disease severity, sex, and medication.

## Discussion

In the current study, the NINCDS-ADRDA criteria was used as an accurate clinical diagnosis of AD was paramount. The NINCDS-ADRDA criteria have been demonstrated as a reliable tool for the diagnosis of probable AD. A review by Knopman *et al*. that included more than a dozen clinical pathological studies found that the sensitivity and specificity of the NINCDS-ADRDA criteria were 81% and 70%, respectively [25]. In this study, 14.2% of the PwAD reported VHs at a once-a-month frequency, which is consistent with the majority of previous studies investigating VHs in AD. The frequency of VHs is much lower in AD than in DLB, based on the studies investigating VH comorbidity in DLB [6–8]. Holroyd *et al*. found that older age, female sex, decreased visual acuity, and presence of visual agnosia were associated with VHs in AD [12]. Impaired visual acuity is highly associated with VHs in AD [12,13]; therefore, patients with uncorrected visual dysfunctions were excluded in this study to avoid confounding. Comparison of patient demographics between the VH+ and VH− groups revealed that VHs in AD were associated with older age and more severe dementia stage, a finding that was also consistent with previous studies [9–13]. Based on this robust evidence, we adjusted age and dementia severity to examine associations between VHs and demographical factors among all AD patients and found that VHs were associated with female gender, longer duration of psychiatric disorders, higher total NPI score, higher caregiver burden score, and higher rates of antipsychotic use. Previous studies failed to reach a consensus or did otherwise fail to report on the frequency of VHs in females [11,12]. Association of more severe neuropsychiatric symptoms revealed in our analysis also deserves attention, because the precise diagnosis and management of VHs using either pharmacological or non-pharmacological methods should be considered to reduce caregiver burden during at any disease stage.

Our findings support the hypothesis that AD with VHs has higher clinical features for the diagnosis of DLB. We cannot exclude the possibility that some of the PwAD with VHs in the current study were actually possible or probable DLB patients, due to the relatively low sensitivity of the clinical diagnostic criteria for DLB. However, compared to the first consensus criteria defined in 1996, the sensitivity of clinical DLB diagnosis has improved greatly with the introduction of the third consensus criteria proposed in 2005. Using these newer criteria, the diagnostic rate of probable or possible DLB was about 16% in our dementia database, all of which were excluded in the current study. Together with previous clinical and pathological studies [17,21,22], we propose that it is highly possible that VHs in AD patients in the current cohort were strongly associated with Lewy body-associated pathology in the brain. Such mixed pathology was not previously reported in AD patients presenting with delusions, although both delusions and VHs are striking psychotic symptoms of AD. Observation of delusions in early-stage AD and VHs in later stages of AD might also be explained by different neuropathologic mechanisms contributing to two different psychotic symptoms during different stages of the disease. Furthermore, as patients with DLB are more sensitive to antipsychotics, their use should be considered with more caution if there is a significant probability of comorbid pathology. Antipsychotics are occasionally given to dementia patients with behavioral and psychological symptoms. Half of the DLB patients treated with antipsychotics might develop severe complications including deterioration of parkinsonian symptoms, altered mental status, fever, and autonomic dysfunction.

Finally, it is critical to note that AD with VHs was associated with higher severity and increased frequency of most NPI subscales including delusions, hallucinations in the non-visual domain, agitation, depression, anxiety, disinhibition, irritation, and aberrant motor behavior. These results are also consistent with previous studies that addressed this issue [15, 17, 33]. Hallucinations in AD patients were found to be significantly correlated with poor inhibition and depression [15]. Many of the neuropsychiatric symptoms are mediated by inhibition, such as depression [17]. Dementia patients with VHs are more likely to exhibit delusions and apathy than those without VHs [17]. VHs in individuals with autopsy-confirmed DLB were previously reported to be associated with personality changes, including apathy [34]. Comparison of the clinical history of VRFs in the current study showed no significant association. However, a history of hypertension or other VRFs alone without true gauge could represent the current and accurate vascular status of the patients. Future studies including larger sample sizes and more detailed evaluation of VRFs are necessary to clarify and confirm the potential association of these factors with VHs in AD. MMSE and CASI were used to assess the cognitive function of patients, which also revealed that there was no association between any of the cognitive domains of MMSE or CASI and VHs in AD. One reason for this outcome might be the lack of sensitivity of the screening cognitive test in differentiating certain cognitive features that are more striking in DLB such as executive function, visuospatial function, and attention.

Our study has several limitations that should be noted. First, this study was conducted at a single hospital in central Taiwan. Therefore, a selection bias cannot be denied, and our findings may not be generalizable to all patients with AD. Future studies should target recruitment of dementia patients from multiple centers to control selection bias. Second, this was a cross-sectional study comparing VH+ and VH− PwAD to determine factors associated with VHs; therefore, a causal relationship of factors with dementia cannot be determined. Third, because of the lack of beta amyloid or dopamine transporter imaging, beta amyloid or Tau measurement in cerebrospinal fluid, or post-mortem pathological evaluation, the diagnosis of AD was based on the clinical criteria for typical AD, which might have resulted in a lower diagnostic accuracy for AD.

In summary, VHs, albeit occurring at a low rate, had a high impact in AD. Diagnostic features of DLB including cognitive fluctuation, RBD, and severe neuroleptic sensitivity were highly associated with VHs in AD. PwAD with VHs tended to have more severe neuropsychiatric symptoms and more caregiver burden.
